# Recalled Adverse Childhood Experiences Predict Behavioral Traits Associated With an Accelerated Life History in Cebu, Philippines

**DOI:** 10.1002/ajhb.70255

**Published:** 2026-04-08

**Authors:** Sofia C. Carrera, Delia B. Carba, Nanette R. Lee, Lee T. Gettler, Christopher W. Kuzawa

**Affiliations:** ^1^ Department of Anthropology Northwestern University Evanston Illinois USA; ^2^ Office of Population Studies Foundation, Inc. University of San Carlos Philippines; ^3^ Department of Anthropology University of Notre Dame Notre Dame Indiana USA; ^4^ Eck Institute for Global Health University of Notre Dame Notre Dame Indiana USA; ^5^ Department of Human Evolutionary Biology Harvard University Cambridge Massachusetts USA

**Keywords:** adverse childhood experiences, childhood maltreatment, early‐life adversity, life history theory, maturation, reproductive strategies

## Abstract

**Objectives:**

Adverse childhood experiences (ACEs) have long been associated with poor health in adulthood, with many researchers interpreting these findings as evidence of a “fast” life history. In this study we utilize data from the Cebu Longitudinal Health and Nutrition Survey (CLHNS) to test the expectations of this framework among inhabitants of the Cebu metropolitan area in the Philippines.

**Methods:**

Data on development, behavior, and reproduction were collected from study participants (*N* = 1288, 54% male) over the course of multiple survey rounds, beginning before their birth in 1983–84. In 2018, participants completed a retrospective ACEs questionnaire. We built discrete hazard models and generalized linear models to test whether recalled ACEs predict characteristics of a “faster” life history in CLHNS.

**Results:**

There was no significant relationship between ACEs and maturational timing, but individuals who recalled more ACEs engaged in health‐risk behaviors earlier and exhibited younger ages at sexual debut. Among women specifically, ACEs also predicted a younger age at first reproduction and higher gravidity. After splitting ACEs into two dimensions, the same results were observed in response to deprivation but not threat.

**Conclusions:**

As in other low resource settings, physical maturation in Cebu was accelerated in households with greater access to resources but not in response to early psychosocial stressors as indicated by recalled ACEs. However, individuals who experienced ACEs did exhibit behavioral profiles consistent with faster life history scheduling and greater investment in reproduction.

## 1 Introduction

Exposure to early‐life adversity (ELA) predicts an increased risk of disease (Galobardes et al. 2006; Huang et al. 2015) and premature mortality (Felitti et al. 1998; Kelly‐Irving et al. 2013; Yu et al. 2022), but why experiences in childhood impact health decades later remains unclear. One popular hypothesis draws on a fundamental theory in evolutionary biology—life history theory—which describes how organisms distribute finite energy between the crucial biological processes of growth, reproduction, and survival. Across species, high extrinsic (unavoidable) mortality risk selects for a “fast” life history that prioritizes reproduction over longevity (Promislow and Harvey 1990), as investment in slowing the pace of aging via somatic maintenance offers little benefit to an individual who is unlikely to reach old age. This framework has been adopted by the social sciences in an effort to explain individual variation in the pace of life history within a species. Originally proposed by Belsky et al. (1991), “psychosocial acceleration theory” argues that ELA serves as a cue of mortality risk and promotes a faster life history via increased investment in reproduction (e.g., earlier maturation, increased mating effort) and decreased investment in long‐term health (e.g., immune function, tissue repair).

Early tests of this hypothesis provided extensive support, particularly in regard to maturational timing (e.g., (Ellis and Garber 2000; Kim et al. 1997; Moffitt et al. 1992)), but more recent work has revealed important caveats including the potentially confounding role of SES and nutrition (Pham et al. 2022; Zhang et al. 2019). A classic example concerns paternal absence, which has been interpreted as signaling familial instability (Belsky et al. 1991; Draper and Harpending 1982). Ample research in high‐income, post‐industrialized countries indicates that girls who grow up without a father present begin investing in reproduction earlier, as evidenced by a younger age at menarche (Holdsworth and Appleton 2020; Nettle 2010; Webster et al. 2014). However, in these energy‐replete societies, single‐parent households are more likely to be living in poverty (Christopher et al. 2002) and low socioeconomic status (SES) is associated with high‐fat, high‐calorie diets. This evolutionarily‐novel characteristic of the environment (Wells 2012) can independently accelerate maturation (Bogaert 2008; Kyweluk et al. 2018; Zhou et al. 2022). In contrast, childhood undernutrition is more prevalent than childhood obesity in many low‐ and middle‐income countries (LMICs), and in such settings, it is not psychosocial stress but higher socioeconomic status and/or favorable nutrition that is consistently linked to earlier maturational timing (Anderson 2015; Gettler et al. 2015; Kyweluk et al. 2018; Sear et al. 2019; Sheppard et al. 2014). This growing body of literature supports later amendments to psychosocial acceleration theory that specify the need for adequate energetics to support accelerated maturation (Coall and Chisholm 2003; Ellis 2004; Ellis et al. 2024).

Data from wild, non‐human primates suggest that energetics, not psychosocial adversity, would have been the primary driver of biodemographic life history traits in early hominins. Low social rank is associated with psychosocial and nutritional stress (Jarvey et al. 2024; Murray et al. 2006; Sapolsky 2021), reduced longevity (Weibel et al. 2020), and lower lifetime reproductive success (Lea et al. 2015). Low‐ranking individuals would theoretically benefit from an accelerated life history that prioritizes early and fast reproduction, but ample evidence shows the opposite. In various primate species, females of low social status delay maturation and/or have a slower reproductive pace (Carrera 2023; Charpentier et al. 2008; Emery Thompson et al. 2016; Feder et al. 2022; Pusey et al. 1997; Weibel et al. 2020). In line with these findings, data from historical human populations link low SES with delayed first reproduction (Pettay et al. 2007) and lower fertility (Hayward et al. 2013; Hayward et al. 2016; Liu and Lummaa 2011; Pettay et al. 2007; Volk 2023), reflecting the energetic requirements of maturation, ovulation, and lactation (Ellison 2003).

Today, humans occupy environments with far greater resource availability and are less likely to face severe caloric deficits (Alt et al. 2022). As a result, the relationship between SES and biodemographic life history traits is rapidly changing. Growth‐related traits like maturational pace remain dependent on energetics in many LMICs (Abioye‐Kuteyi et al. 1997; Belachew et al. 2011; Bosch et al. 2008; Chowdhury et al. 2000; Odongkara Mpora et al. 2014), but the behavioral dimensions of reproductive strategies are less constrained by resource access and more susceptible to ELA (Ellis et al. 2024; Gettler et al. 2015; Kyweluk et al. 2018). Indeed, although energy‐rich populations may be capable of shorter interbirth intervals (Sadhir and Pontzer 2023), in practice low SES is often associated with greater fertility in energy‐stressed populations (Lai and Tey 2014; Sheppard et al. 2016), mirroring the patterns typical of high‐income countries like the US (Dribe et al. 2014).

Other life history‐related behaviors like risk‐taking are even less likely to depend on resource access, and they are consistently increased in response to psychosocial adversity. Social scientists posit that risk‐taking propensity reflects a prioritization of current reproductive opportunities over long‐term health and relationships, while risk‐aversion reflects an investment in future health and long‐term mating strategies (e.g., monogamous pair bonds, biparental care) characteristic of a “slow” life history strategy (Cabeza de Baca and Ellis 2017; Wang et al. 2009; Wolf et al. 2007). For instance, having more sexual partners and engaging in extra‐pair mating can maximize reproductive output and genetic quality (Gangestad and Simpson 2000) but also increase the risk of contracting sexually‐transmitted infections (Hillis et al. 2000). Evolutionary psychology has also focused on adolescent health‐risk behaviors which increase the likelihood of later substance dependence (Donoghue et al. 2017; Hingson et al. 2006; Takakura and Wake 2003). Engaging in such behaviors may reflect a discounting of potential future consequences for individuals who expect a short lifespan (McDade et al. 2011; Vincke 2017; Wang et al. 2009), and in some settings smoking and drinking increases attractiveness to the opposite sex (Richardson et al. 2017; Vincke 2016, 2017). Across diverse ecological contexts, psychosocial stress and other cues of mortality have been associated with these behavioral characteristics of a “fast” life history (Anderson 2015; Kappel et al. 2021; Meeker et al. 2021; Ramiro et al. 2010; Richardson et al. 2018; Shenk et al. 2013; Waynforth et al. 1998), even in populations where maturation is constrained by energetics (Gettler et al. 2015; Hillis et al. 2001; Kyweluk et al. 2018; Ramiro et al. 2010; Richardson et al. 2020).

Different dimensions of ELA can also affect life history pacing in distinct ways (Dinh et al. 2022; Ellis et al. 2022). The Adverse Childhood Experiences (ACEs) scale was developed to reflect several different categories of adversity, such as physical abuse (e.g., being hit) or household dysfunction (e.g., parental separation) (Felitti et al. 1998). Cumulative ACEs reflects the *sum* of these different types and predicts a greater likelihood of health‐risk behaviors (e.g., smoking, alcohol use) (Hughes et al. 2017), cardiovascular disease (Felitti et al. 1998), and premature mortality (Kelly‐Irving et al. 2013), making this scale a valuable tool for medical practitioners. However, this approach assumes that all types of adversity have equal and additive effects (Dinh et al. 2022). McLaughlin and Sheridan (2016) have described two dimensions of ELA that exert distinct effects on biological and neurological development: threat and deprivation. Experiences of *threat* pose direct harm to the child and/or their survival (e.g., physical abuse) and have been associated with earlier maturation (Colich et al. 2020; Sumner et al. 2019), earlier sexual debut (Thomas et al. 2023), and accelerated reproduction (Sanchez‐Cespedes 2018; Weitzman et al. 2023; Yuan et al. 2022). In contrast, experiences of *deprivation* reflect a lack of critical environmental inputs for development, such as emotional neglect or insufficient nutrition, and may actually serve as a constraint to physical maturation and other biodemographic life history traits (Jørgensen et al. 2023; Sumner et al. 2019; Yuan et al. 2022).

Here, we examine the relationship between recalled ACEs and the pace of life history in a well‐characterized longitudinal cohort, the Cebu Longitudinal Health and Nutrition Survey (CLHNS). This unique birth cohort study began in 1983 in Metro Cebu, Philippines, providing rare prospective data on the timing of life history‐related traits. This study population did not grow up with the potential confound of evolutionarily‐novel high‐calorie diets. Rather study participants faced high rates of stunting (Adair and Guilkey 1997) and low rates of obesity as children (Dahly et al. 2013), and the community exhibits a positive association between SES and BMI (Dahly et al. 2010). Prior work from Cebu has supported the expectation that earlier maturation is tied to favorable childhood nutrition, not mortality cues (Gettler et al. 2015; Kyweluk et al. 2018), and here we expand upon these findings by using recalled ACEs as a proxy for ELA. We examine the effects of the traditional cumulative ACEs score and scores for two distinct dimensions: threat and deprivation. In the context of Cebu, we predict that earlier male and female maturation will be more strongly associated with high early‐life SES than with exposure to adversity. Although female reproductive success also requires sufficient energetic resources (Ellison 2003), it is likely that only severe caloric deficits result in delayed or slower reproduction (e.g., high‐performance athletes (Boutari et al. 2020)). We do not expect energetic conditions in Cebu to be sufficient to observe these constraints, leading to our prediction that behavioral dimensions of accelerated reproductive strategies (i.e., earlier first reproduction, higher gravidity) will be associated with recalled ACEs, mirroring prior findings on the transition to fatherhood in Cebu (Gettler et al. 2015). Behavioral life history‐related traits should not be constrained by energetics, so we predict that recalled ACEs will be associated with an earlier onset of these behaviors: smoking, drinking, romantic relationship, and sexual debut. Finally, we expect to find minimal correlations between the biodemographic and the behavioral outcomes.

## 2 Materials and Methods

### 2.1 Study Participants

Data for this analysis come from the Cebu Longitudinal Health and Nutrition Survey (CLHNS), an ongoing, long‐term birth cohort study of 3080 mothers and their children born in Cebu, Philippines between 1983 and 1984. Mothers in their third trimester of pregnancy were recruited via a random clustering sampling procedure from 33 different communities (17 urban, 16 rural) in the metropolitan Cebu area. Initial and follow‐up surveys were conducted via question‐based, at‐home interviews. These analyses utilize data from multiple surveys conducted between 1983 and 2018. All participants who had complete data on ACEs and biodemographic traits of life history were included (*N* = 1288 (54% male)). All data were collected under conditions of informed consent with institutional review board approval from Northwestern University, University of North Carolina—Chapel Hill, and University of San Carlos, Cebu, Philippines.

### 2.2 Recalled Adverse Childhood Experiences (ACEs)

In 2018 a questionnaire was administered to the CLHNS participants (mean age 35 years old) to determine the prevalence of ACEs in this population. This questionnaire (translated into the local Cebuano dialect) contained 19 individual questions about specific adverse experiences (Cronbach's alpha = 0.82, CI = (0.79, 0.84)). Each participant recalled whether or not they had experienced each item during their first 18 years of life.

#### 2.2.1 Cumulative ACEs

**FIGURE 1:**
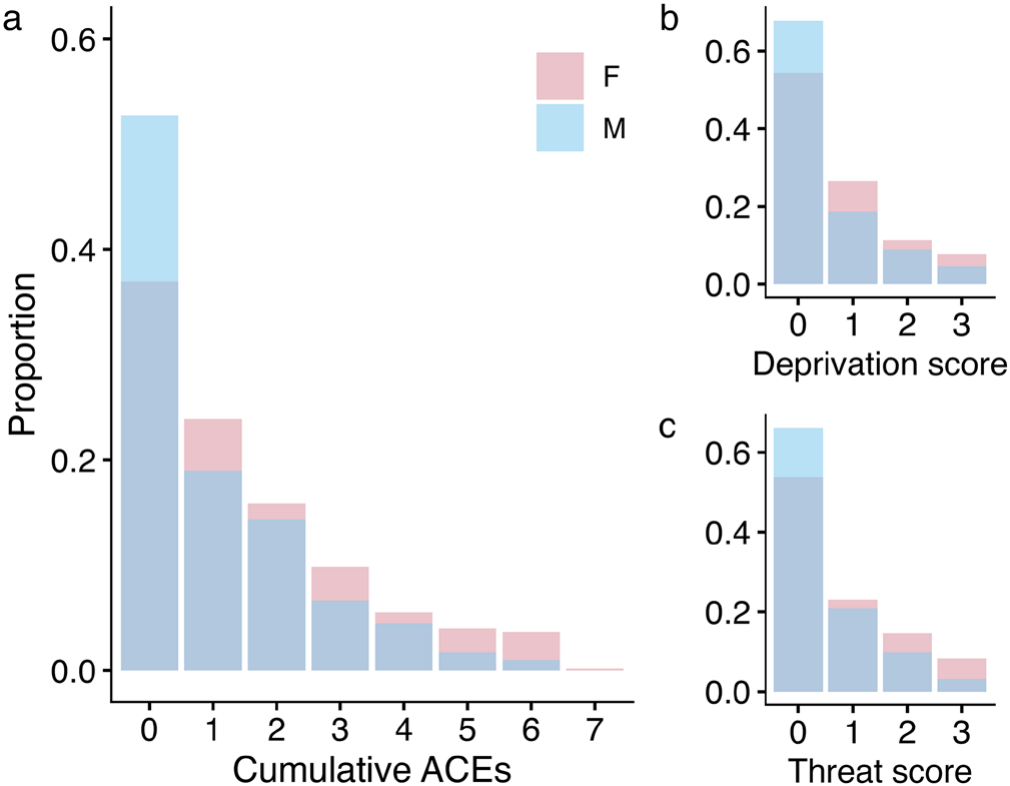
Proportion of female (pink) and male (blue) study participants reporting ACEs. (a) Cumulative ACEs, (b) deprivation score, and (c) threat score.

The questionnaire covered seven distinct subtypes of adversity: emotional abuse, physical abuse, sexual abuse, emotional neglect, physical neglect, interpersonal violence, and household dysfunction (Felitti et al. 1998; Sumner et al. 2019) (Table A.1). We calculated a “cumulative ACEs” score for each participant as the number of unique categories that an individual experienced (range: 0–7) (Figure 1a).

#### 2.2.2 Dimensions of ACEs

We binned the seven ACE categories into two dimensions following previous studies (Henry et al. 2021; Sumner et al. 2019). Threat consisted of interpersonal violence, emotional, physical, and sexual abuse, and deprivation consisted of household dysfunction, emotional and physical neglect (Table A.1). A cumulative score was calculated for each dimension as the number of categories that a participant experienced (Sumner et al. 2019) (Figure 1b,c). As expected, reports of deprivation experiences, but not threat, were associated with low SES in adolescence (Figures A.1 and A.2).

### 2.3 Covariates

Data for control variables were retrieved from the baseline survey (1983). Two variables were used to reflect early‐life SES. Household assets were scored (0–9) based on several variables, including home ownership, electricity in the home, housing material type, and ownership of items such as an air conditioner, television, refrigerator, or car. Maternal education level was based on the number of years of completed education. Mother's recalled age at menarche was also recorded at baseline and included in these analyses to partially adjust for genetic confounds regarding the pace of life history (Kyweluk et al. 2018).

Compared to members of the original birth cohort who were not part of this study (*N* = 1792), the analytical sample (*N* = 1288) had lower SES at baseline: fewer years of maternal education (7.17 vs. 7.84, *p* < 0.001) and fewer household assets (2.41 vs. 2.59, *p* = 0.06).

### 2.4 Outcome Variables

#### 2.4.1 Biodemographic Life History Traits

Outcomes related to life history trajectories in females were age at menarche, age at first conception, and gravidity by 2018 (mean age 35 years old). At approximately 11.5, 14.5, 19.5, and 22 years old, female participants were asked if they had experienced menses, and if so, at what age. In each adult survey round, females updated their reproductive histories with the duration and termination date of each new pregnancy. Age at first conception was calculated by subtracting the reported pregnancy duration from age at first pregnancy termination, allowing us to adjust for premature births or miscarriages. The number of pregnancies (regardless of outcome) a woman had by their interview in 2018 was recorded as their gravidity.

For male life histories, we assessed self‐reported Tanner stage in 1998 (mean 14 years old). Participants rated themselves on a scale of 1 (least developed) to 5 (most developed) in comparison to drawings of the pubic region. Because a key goal is to use directly comparable measures in males and females, we did not assess age at first reproduction in males as these reports are potentially less reliable than in females, especially in a setting like Cebu where there is cultural emphasis on two‐parent households (Gettler et al. 2022). See Gettler and colleagues (Gettler et al. 2015) for an analysis of the transition to fatherhood in the CLHNS.

#### 2.4.2 Behavioral Life History‐Related Traits

When participants were approximately 14.5, 19.5, 22, and 35 years old, they retrospectively reported the age at which they first engaged in four life history‐related behaviors. We used the age reported at the earliest survey to reduce recall bias. Specifically, participants reported the age at onset of (i) smoking, (ii) drinking alcohol, (iii) entering a romantic relationship, and (iv) sexual debut. If an individual reported never having engaged in a behavior, they were right‐censored at the age of the 2018 interview for that outcome variable.

### 2.5 Statistical Analyses

All analyses were run using R Statistical Software (v4.5.2; R Core Team 2025). We first built generalized linear models (GLMs) using the ‘glmmTMB’ package (v.1.1.7) (Brooks et al. 2017) to predict the number of recalled ACEs from baseline SES (maternal education and household assets in 1983) and participant sex (assigned at birth). Due to overdispersion, cumulative ACEs were modeled with a zero‐inflated negative binomial distribution while threat and deprivation scores were each modeled with a zero‐inflated Poisson distribution. Model diagnostics were assessed using the “DHARMa” package (v.0.4.6) (Hartig 2020). We then examined Spearman correlations between the outcome variables for males and females separately.

Survival analyses were conducted to predict age at menarche and age at first conception in females, as well as the age at onset of the four life history‐related behaviors (initiation of smoking, drinking, romantic relationship, and sexual intercourse) in both sexes. Age at each outcome was reported in years, so we built discrete hazard models (Tutz and Schmid 2016) using the “discSurv” package (v.2.0.0) (Welchowski et al. 2022). Males had an earlier age of onset for all behaviors except first romantic relationship (Figure A.3), leading us to model the sexes separately.

We built GLMs using the “glmmTMB” package to predict gravidity (in females) and pubertal development (in males). Gravidity was modeled using a zero‐inflated negative binomial distribution and controlling for age at the 2018 interview. In males, a binary variable was created to reflect advanced pubertal development (Tanner score 4 or 5) and modeled using a binomial distribution, controlling for age at the 1998 interview when Tanner score was reported. Model fit was assessed using the ‘DHARMa’ package.

We built two primary survival models for each biodemographic and behavioral outcome. The first used cumulative ACEs (coded as 0, 1, 2, or 3+ (Iob et al. 2020; Merrick et al. 2018)) as the sole predictor; the second used threat scores and deprivation scores (coded as 0, 1, 2, or 3+) as the predictors. Next, two adjusted models were built by adding control variables (household assets, maternal education, and maternal age at menarche) to the primary models.

## 3 Results

### 3.1 ACEs In CLHNS

Of the 1288 participants included in these analyses, 55% (*N* = 703) recalled at least one adverse experience prior to age 18 years. Overall, participants were equally likely to report at least one type of experience related to threat (40%) or deprivation (38%), but male participants reported significantly fewer cumulative ACEs (*p* < 0.05, Table 1) and had lower threat scores (*p* < 0.01, Table A.2) compared with female participants. Male sex also predicted “excess” zeros for cumulative ACEs and deprivation scores, as noted via the zero‐inflated formula output for these models. Somewhat unexpectedly, participants born into households with greater assets at baseline had higher cumulative ACEs (Table 1) and threat scores (Table A.2). This positive association is potentially related to links between household structure and later family experiences in Cebu. Children born into multi‐nuclear households had higher SES at baseline, but they were also more likely to later report adverse experiences such as household dysfunction (35%) and physical abuse (22%) compared with those born into extended family (18% and 19%, respectively) or single nuclear family (24% and 18%, respectively) households.

**TABLE 1:** Results from a zero‐inflated negative binomial model predicting the number of cumulative ACEs a participant reported based on baseline SES variables and participant sex (assigned at birth). Participant sex was used as a predictor of zero‐inflation (ZI).

Predictor	Cumulative ACEs			
	IRR^a^	95% CI^b^	*p*	
Household assets	1.07	[1.00, 1.15]	**0.044**	*
Maternal education	1.02	[0.95, 1.10]	0.605	
Male	0.83	[0.71, 0.96]	**0.013**	*
*ZI formula*	Coef.	95% CI^b^	*p*	
Male	2.26	[1.41, 3.60]	**< 0.001**	***

*Note:* Bold values indicate significant *p*‐values.

^a^ IRR, incidence rate ratio.

^b^ CI, confidence interval.

### 3.2 Life History‐Related Outcomes

**FIGURE 2:**
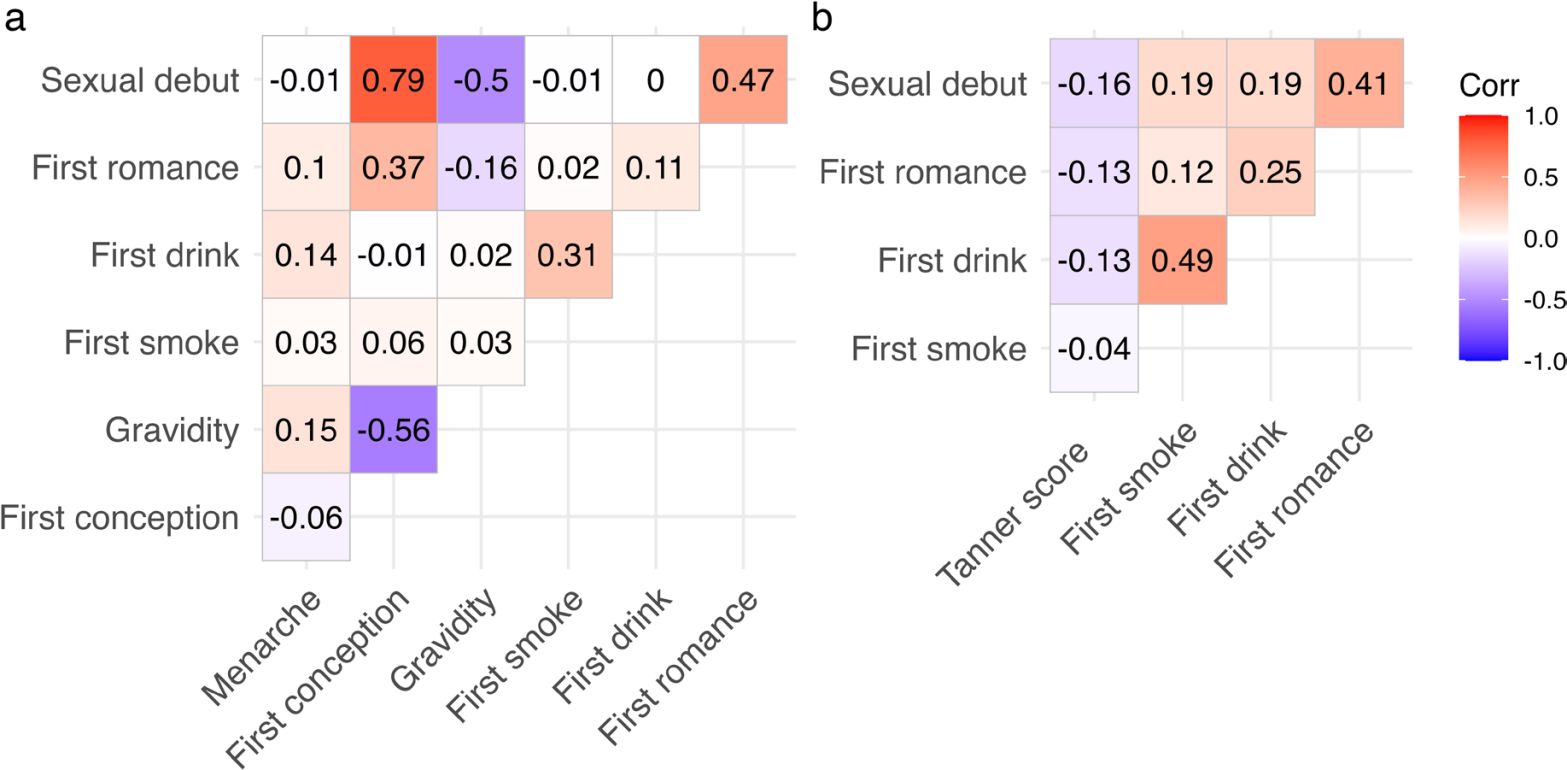
Spearman correlation matrices for the life history‐related outcomes in (a) females and (b) males. In males, pubertal development was assessed via self‐rated Tanner scores ranging from 1 (least developed) to 5 (most developed). It should be noted that we did not adjust for male age when Tanner score was reported (range: 15.3–16.9 years).

Older age at menarche was weakly correlated with increased gravidity, later onset of drinking, and older age at first romantic relationship (Figure 2a), but there was no association between age at menarche and age at sexual debut, first conception, or first smoke. Accelerated pubertal development in males exhibited consistent but weak associations with an earlier onset of life‐history related behaviors (Figure 2b). In females the strongest correlation was between age at sexual debut and age at first conception, such that those who had sex at a younger age also conceived earlier (*r* = 0.79). Earlier sexual debut and earlier first conception were also each associated with higher gravidity by age 35 years (*r* = −0.50 and −0.56, respectively).

#### 3.2.1 Biodemographic Life History Traits

**FIGURE 3:**
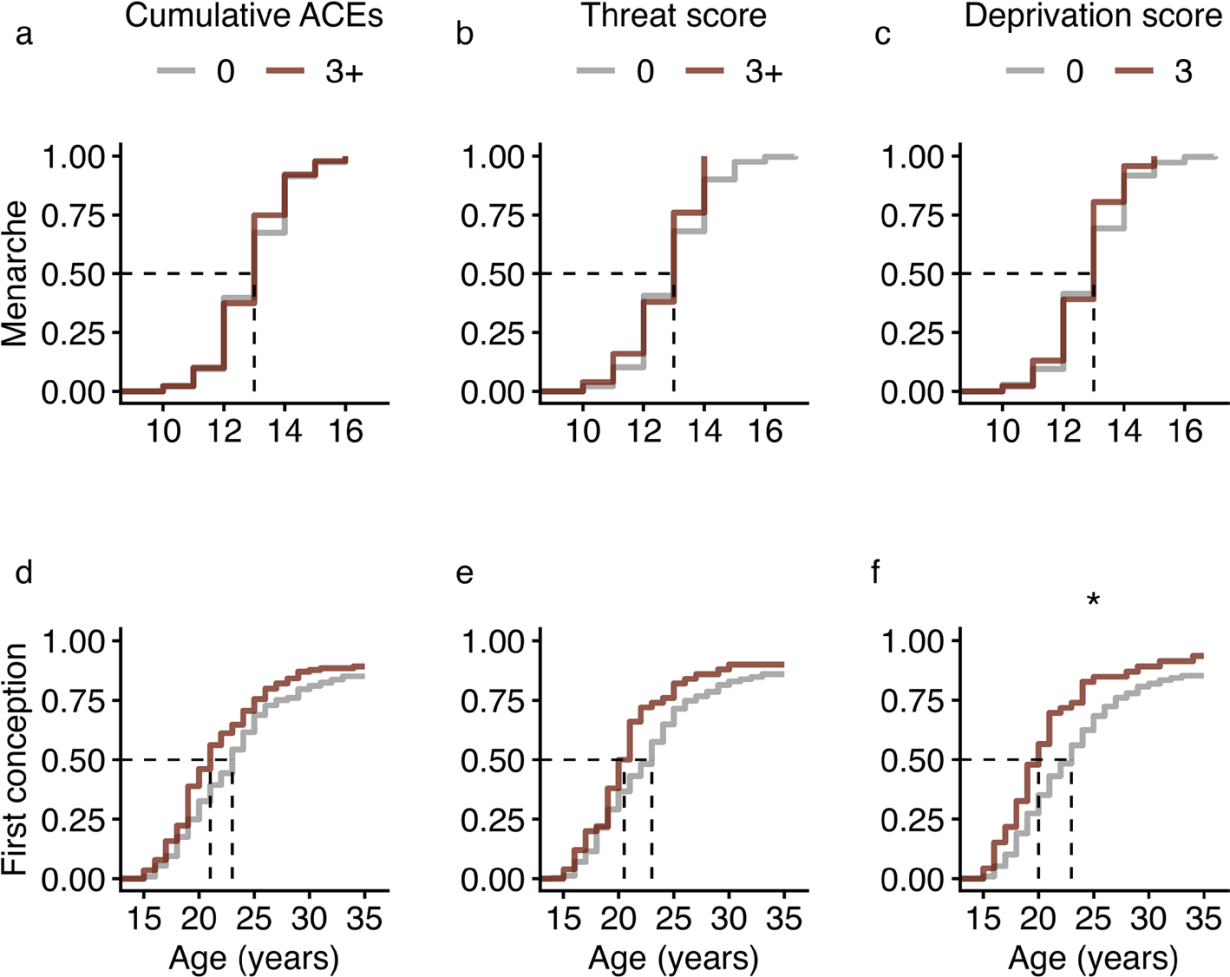
Cumulative event plots for the primary (unadjusted) models predicting age at menarche (top row) and age at first conception (bottom row) in females. Gray lines reflect participants reporting 0 ACEs. Red lines reflect participants reporting 3+ ACEs. The dotted black lines perpendicular to the axes indicate the age by which 50% of each group had experienced the outcome of interest. Note the different *x*‐axes for visualization purposes.

Maturational timing was not predicted by any of our proxies for ELA (cumulative ACEs, threat scores, deprivation scores) in females (Figure 3a–c) or in males (Table 2). These results remained consistent in the adjusted models which revealed that higher maternal education at baseline predicted earlier maturation in both sexes (Tables A.3 and A.4). In females, younger age at first conception was predicted by higher deprivation scores (Figure 3f) and exhibited a trending relationship with more cumulative ACEs. Higher female gravidity was also predicted by cumulative ACEs and deprivation scores (both *p* < 0.05), but not by threat scores (Table 2). Results were robust to adjustment for SES, except the association between deprivation and gravidity weakened (Table A.3). Higher maternal education at baseline predicted a later age at first conception and lower gravidity (Table A.3).

**TABLE 2:** Coefficient estimates for the predictors of interest in the unadjusted models predicting biodemographic life history traits in females and males. Discrete hazard models (Gompertz) were run for maturation and first conception in females, and the coefficients reflect the hazard ratio (HR). Generalized linear models (zero‐inflated negative binomial) were run for gravidity and the coefficient reflects the incidence rate ratio. Generalized linear models (binomial distribution) were run for early pubertal development in males and the coefficient reflects the odds ratio.

Outcome	Predictor	Females			Males		
		Coef.	95% CI^a^	*p*	Coef.	95% CI^a^	*p*
Maturation	Cumulative ACEs	1.02	[0.95, 1.09]	0.552	1.16	[1.00, 1.35]	0.058
	Threat score	1.07	[0.98, 1.18]	0.139	1.14	[0.97, 1.35]	0.109
	Deprivation score	0.98	[0.89, 1.08]	0.679	1.00	[0.85, 1.19]	0.957
First conception	Cumulative ACEs	1.07	[0.99, 1.15]	0.078			
	Threat score	1.02	[0.93, 1.13]	0.651			
	Deprivation score	**1.12**	**[1.01, 1.24]**	**0.036**			
Gravidity	Cumulative ACEs	**1.06**	**[1.01, 1.12]**	**0.026**			
	Threat score	1.02	[0.96, 1.08]	0.579			
	Deprivation score	**1.06**	**[1.00, 1.13]**	**0.048**			

*Note:* Bold values indicate significant *p*‐values.

^a^ CI, confidence interval.

#### 3.2.2 Behavioral Life History‐Related Traits

**FIGURE 4:**
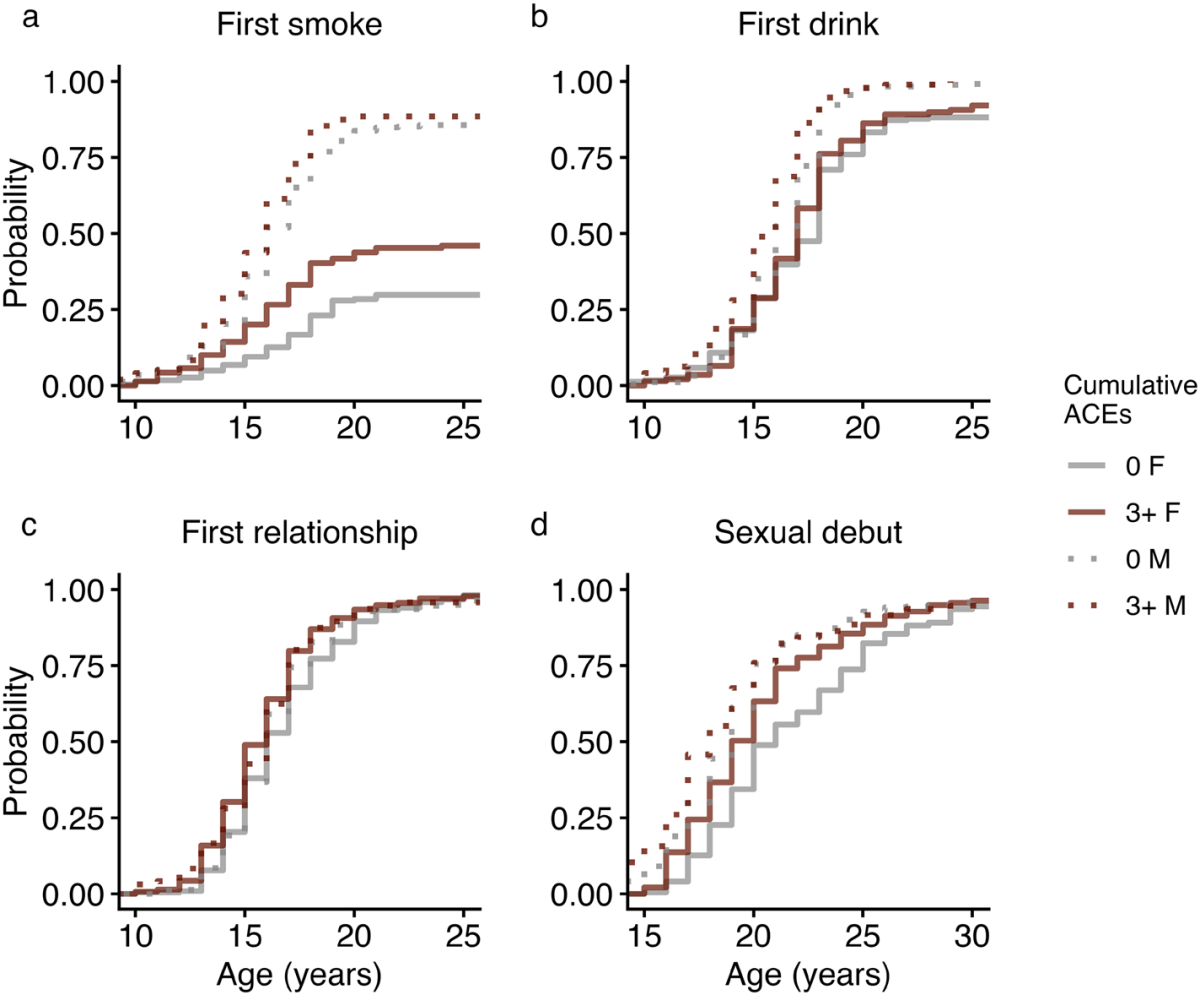
Cumulative event plots for the primary (unadjusted) models predicting the onset of behavioral life history‐related traits in females (solid lines) and males (dashed lines): (a) first smoke, (b) first drink, (c) first romantic relationship, and (d) sexual debut. Gray lines reflect participants reporting 0 cumulative ACEs. Red lines reflect participants reporting 3+ cumulative ACEs. Note the different *x*‐axes for visualization purposes.

In females, younger age at first smoking was predicted by cumulative ACEs (Figure 4a) and threat scores (both *p* < 0.01) while age at first drink was not associated with ACEs (Table 3). Males exhibited the opposite pattern: none of our proxies of ELA were associated with age at first smoking, but a younger age at first drink was predicted by cumulative ACEs (*p* < 0.01) and deprivation scores (*p* < 0.05) (Figure 4a,b). In both sexes, deprivation scores predicted an earlier sexual debut, and the same relationship was observed with cumulative ACEs in females (all *p* < 0.05). In neither sex were ACEs associated with age at first romantic relationship (Figure 4c,d). These results were robust to adjustment for SES (Tables A.5 and A.6). Higher SES predicted an earlier age at first drink but a later age at first relationship and sexual debut in females, while higher SES was associated with a later age at first smoking and an earlier sexual debut in males.

**TABLE 3:** Coefficient estimates for the predictors of interest in the unadjusted models predicting the onset of behavioral life history‐related traits. Discrete hazard models (Gompertz) were run separately for males and females and for each outcome of interest.

Outcome	Predictor	Females			Males		
		HR^a^	95% CI^b^	*p*	HR^a^	95% CI^b^	*p*
First smoke	Cumulative ACEs	**1.17**	**[1.05, 1.31]**	**0.005**	1.05	[0.97, 1.13]	0.229
	Threat score	**1.22**	**[1.06, 1.41]**	**0.006**	1.04	[0.93, 1.16]	0.458
	Deprivation score	1.09	[0.93, 1.26]	0.273	1.04	[0.93, 1.15]	0.503
First drink	Cumulative ACEs	1.04	[0.97, 1.11]	0.312	**1.10**	**[1.03, 1.18]**	**0.007**
	Threat score	1.09	[0.99, 1.20]	0.071	1.01	[0.91, 1.12]	0.859
	Deprivation score	0.96	[0.87, 1.06]	0.421	**1.11**	**[1.01, 1.23]**	**0.034**
First relationship	Cumulative ACEs	1.07	[0.99, 1.14]	0.071	1.03	[0.96, 1.10]	0.399
	Threat score	1.07	[0.97, 1.17]	0.158	0.98	[0.88, 1.09]	0.741
	Deprivation score	1.05	[0.96, 1.15]	0.307	1.08	[0.98, 1.19]	0.107
Sexual debut	Cumulative ACEs	**1.11**	**[1.04, 1.19]**	**0.003**	1.01	[0.94, 1.09]	0.688
	Threat score	1.06	[0.96, 1.16]	0.250	0.94	[0.85, 1.04]	0.258
	Deprivation score	**1.13**	**[1.02, 1.24]**	**0.015**	**1.11**	**[1.00, 1.23]**	**0.039**

*Note:* Bold values indicate significant *p*‐values.

^a^ HR, hazard ratio.

^b^ CI, confidence interval.

## 4 Discussion

These results largely support our predictions about the relationship between recalled ACEs and life history‐related biodemographic and behavioral profiles in the CLHNS. In a context where low SES is coupled with reduced energetic resources, we found no significant or meaningful relationships between maturational timing and cumulative ACEs, threat, or deprivation scores. Rather, as previously found in Cebu, earlier maturation was linked with higher SES in both males and females (Gettler et al. 2015; Kyweluk et al. 2018). More recalled ACEs did predict a younger age at sexual debut in both sexes and higher gravidity in females, consistent with the hypothesis that ELA motivates a faster life history strategy.

Individuals who recalled more ACEs exhibited greater risk‐taking propensity regardless of childhood SES, but associations differed between the two dimensions of ACEs. Threat scores predicted an earlier onset of smoking in females, while deprivation scores predicted an earlier first drink in males. In both sexes, only deprivation was associated with an earlier sexual debut, complementing prior findings from Cebu linking paternal instability to earlier sexual debut and earlier transition to fatherhood in males, independent of early‐life energetics and maturational timing (Gettler et al. 2015; Gettler et al. 2025). We also observed sex differences in susceptibility and/or response to early SES and recalled, likely due to cultural norms. Apart from maturational timing, greater maternal education at baseline generally predicted characteristics of a “slower” life history in females, similar to results from energy‐replete populations (Hopcroft 2006; Huber et al. 2010; Sheppard et al. 2016). In males, the associations with maternal education were less consistent, with greater maternal education predicting an earlier onset of health‐risk behaviors but a later onset of romantic and sexual behavior. Additionally, while we observed the largest effect sizes of ACEs on smoking in females, there was no relationship between ACEs and smoking in males. In Cebu, most males begin smoking by age 20, but fewer than 50% of females ever reported smoking, suggesting that smoking is a culturally normative and peer‐driven phenomenon for men but more susceptible to psychosocial stress in women.

Given the importance of energetic resources to growth‐related life history traits in Cebu (Gettler et al. 2015; Kyweluk et al. 2018), we were unsurprised that pubertal timing was not meaningfully correlated with behavioral life history‐related traits (Marco Del Giudice 2020). However, we expected age at menarche to be moderately associated with the other biodemographic traits (Ibitoye et al. 2017). Instead, age at first conception and gravidity were highly correlated with age at sexual debut. And unlike maturation, earlier first conception and higher gravidity were predicted by low SES, more cumulative ACEs, and higher deprivation scores, matching predictions of recent models (Ellis et al. 2024) and our prior observations at Cebu (Kyweluk et al. 2018). Female reproductive success may require a threshold level of energetic condition, but once that is met, at least in Cebu, it is driven by behavioral life history‐related traits. These results support the idea that there is not just one continuum of “fast‐slow” life history‐related traits but multiple ways of accelerating life history (Marco Del Giudice 2020), each sensitive to different socio‐ecological variables (Dinh et al. 2022).

Unique features of household structure in Cebu may explain why greater assets at birth predicted more recalled ACEs. The ACEs scale was developed in the US (Felitti et al. 1998) where multi‐family living is more prevalent among low SES populations (Cross 2018; Crouch et al. 2019), linking these household structures to a greater risk of ACEs, such as poverty, paternal absence, and violence (Crouch et al. 2019). In contrast, multi‐family households in Cebu had higher SES than other household types, but participants from these households still recalled more ACEs, such as household dysfunction and physical abuse, likely due to the increased risk of child maltreatment when living with unrelated adults.

Our study has limitations that merit discussion. The use of recall data can introduce bias, and in the case of ACEs, retrospective reports may be only modestly correlated with prospective measures of childhood adversity (Baldwin et al. 2019). Even so, retrospective and prospective ACEs data both predict poor health outcomes in adulthood (Naicker et al. 2021; Reuben et al. 2016). Moreover, the ACEs scale was designed to be administered to adults (Felitti et al. 1998) and is widely and routinely used in the literature to reconstruct ELA, allowing for comparability of research across a broad range of socio‐ecological contexts. In this study, we did conduct a simple validation of recalled ACEs by comparing specific questions to a prospective measure of ELA, household assets during adolescence. As expected, individuals reporting experiences of deprivation lived in households with fewer assets (Figures A.1–A.2).

It is possible that particular types of adversity were underreported in this study. Rates of childhood sexual abuse range between 3% and 31% worldwide (Barth et al. 2013; Pan et al. 2021), and although prevalence was 12% in another Filipino population (Ramiro et al. 2010) it was < 2% in this study. We also lacked data on the exact timing of adverse experiences, leaving open the possibility for certain outcome variables (e.g., maturational timing) to precede exposure to specific adverse events. Nonetheless, our study benefited from the prospective data collection of life history outcomes in Cebu, a rarity in decades long cohort studies. Lastly, heritability plays a role in childhood maltreatment (Dahoun et al. 2025), risk‐taking behaviors (Anokhin et al. 2009), and pubertal timing (Dick et al. 2001; Kirk et al. 2001). Genetic confounds can produce spurious correlations between these variables (Barbaro et al. 2017) or result in an overestimation of the contribution of recalled ACEs to life history pacing (Barbaro et al. 2017). We adjusted for maternal age at menarche in this study to help account for some heritability, but future studies of ACEs and life history would benefit from the incorporation of genetic data.

## 5 Conclusion

Whether evolutionary life history theory, which was developed to explain variation *across* species, can be applied *within* species is hotly debated (e.g., (Sear 2020; Stearns and Rodrigues 2020)). In Cebu we found an association between recalled ACEs and some characteristics of a “faster” life history, specifically via facultative, behavioral components of reproductive strategies, highlighting the role of behavior in driving allocation strategies and impacting reproductive outcomes (Marco Del Giudice 2020). These findings support the recent call for researchers to use the timing of sexual debut, rather than puberty, as a central marker of life history pacing in humans (M. Del Giudice 2025). Criticisms of psychosocial acceleration theory remain, such as the role of density‐dependent competition (André and Rousset 2020; Del Giudice, 2020); but as the field of ELA research evolves, our study exemplifies the benefits of moving beyond the cumulative ACEs score and testing predictions of life history theory in diverse ecological contexts.

## Author Contributions

**Sofia C. Carrera:** conceptualization; methodology; formal analysis; visualization; funding acquisition; writing – original draft. **Delia B. Carba:** data collection; writing – review and editing. **Nanette R. Lee:** data collection; writing – review and editing. **Lee T. Gettler:** data collection; writing – review and editing. **Christopher W. Kuzawa:** conceptualization; data collection; funding acquisition; supervision; writing – original draft.

## Funding

This work was supported by the Bill and Melinda Gates Foundation, Seattle, WA (OPP1164115), the National Institutes of Health (R01 AG061006), and the National Science Foundation (2404774).

## Ethics Statement

This research was conducted with informed consent and human subject clearance from the institutional review boards of Northwestern University, University of San Carlos, and the University of North Carolina. All participants provided written informed consent.

## Conflicts of Interest

The authors declare no conflicts of interest.

## Supporting information


**Table A.1:** Questions included in the ACEs questionnaire and their prevalence in CLHNS. Questions were assigned to seven distinct categories from which cumulative ACEs was calculated, and then categories were binned into two dimensions of adversity to calculate threat and deprivation scores.
**Table A.2:** Model output for generalized linear models (Poisson) predicting threat and deprivation scores from baseline SES variables and participant sex (assigned at birth). Participant sex was used as a predictor of zero‐inflation (ZI). ^†^IRR = incidence rate ratio. ^‡^CI = confidence interval.
**Table A.3:** Results from the adjusted models predicting biodemographic life history traits in females. Discrete hazard models (Gompertz) were run for menarche and first conception, and the coefficients reflect the hazard ratio (HR). Generalized linear models (zero‐inflated Poisson) were run for gravidity and the coefficient reflects the incidence rate ratio. ^†^CI = confidence interval.
**Table A.4:** Results from the adjusted models predicting advanced pubertal development in males. Generalized linear models were run with a binomial distribution and the coefficient reflects the odds ratio. CI = confidence interval.
**Table A.5:** Results from the adjusted models predicting the onset of behavioral life history‐related traits in females. Discrete hazard models (Gompertz) were run for each outcome of interest. ^†^HR = hazard ratio. ^‡^CI = confidence interval.
**Table A.6:** Results from the adjusted models predicting the onset of behavioral life history‐related traits in males. Discrete hazard models (Gompertz) were run for each outcome of interest. ^†^HR = hazard ratio. ^‡^CI = confidence interval.
**Figure A.1:** Socioeconomic status in adolescence and whether an individual reported ACEs related to (a) threat or (b) deprivation. *p* values are from a one‐sided Wilcoxon signed rank test based on the prediction that individuals exposed to ACEs would be from households of lower SES.
**Figure A.2:** Socioeconomic status in adolescence and whether an individual reported experiencing physical neglect. (a) Often felt that they did not have enough to eat, (b) often felt that they had to wear dirty clothes, (c) often felt that their parents were too drunk or high to care for them, (d) had a household member go to prison. *p* values are from one‐sided Wilcoxon signed rank tests based on the prediction that individuals exposed to physical neglect would be from households of lower SES.
**Figure A.3:** Distributions of the age at onset for risk‐taking behaviors in females (pink) and males (blue). (a) first smoke, (b) first drink, (c) first romantic relationship, and (d) sexual debut. *p* values are from two‐sided Wilcoxon signed rank tests.

## Data Availability

Anonymized data and code used in these analyses are publicly available at Github (https://github.com/scarrera11/AJHB_ACEs_LH_March2026) and have been archived within the Zenodo repository (Carrera 2026).
