# Supplementary material for: Recalled Adverse Childhood Experiences Predict Behavioral Traits Associated With an Accelerated Life History in Cebu, Philippines
Source: Am J Hum Biol. 2026 Apr 8;38(4):e70255. doi: 10.1002/ajhb.70255 (PMC13058877; doi:10.1002/ajhb.70255)
Supplement: Supplementary file 1 — Table A.1: Questions included in the ACEs questionnaire and their prevalence in CLHNS. Questions were assigned to seven distinct categories from which cumulative ACEs was calculated, and then categories were binned into two dimensions of adversity to calculate threat and deprivation scores. Table A.2: Model output for generalized linear models (Poisson) predicting threat and deprivation scores from baseline SES variables and participant sex (assigned at birth). Participant sex was used as a predictor of zero‐inflation (ZI). †IRR = incidence rate ratio. ‡CI = confidence interval. Table A.3: Results from the adjusted models predicting biodemographic life history traits in females. Discrete hazard models (Gompertz) were run for menarche and first conception, and the coefficients reflect the hazard ratio (HR). Generalized linear models (zero‐inflated Poisson) were run for gravidity and the coefficient reflects the incidence rate ratio. †CI = confidence interval. Table A.4: Results from the adjusted models predicting advanced pubertal development in males. Generalized linear models were run with a binomial distribution and the coefficient reflects the odds ratio. CI = confidence interval. Table A.5: Results from the adjusted models predicting the onset of behavioral life history‐related traits in females. Discrete hazard models (Gompertz) were run for each outcome of interest. †HR = hazard ratio. ‡CI = confidence interval. Table A.6: Results from the adjusted models predicting the onset of behavioral life history‐related traits in males. Discrete hazard models (Gompertz) were run for each outcome of interest. †HR = hazard ratio. ‡CI = confidence interval. Figure A.1: Socioeconomic status in adolescence and whether an individual reported ACEs related to (a) threat or (b) deprivation. p values are from a one‐sided Wilcoxon signed rank test based on the prediction that individuals exposed to ACEs would be from households of lower SES. Figure A.2: Socioeconomic status in ado [file AJHB-38-e70255-s001.docx]

| **Questionnaire item** | | **Prevalence (%)** |
| --- | --- | --- |
| **Threat** | |  |
| Emotional abuse | |  |
|  | Did a parent or other adult in the household often act in a way that made you afraid that you might be physically hurt? | 28.1 |
|  | Did a parent or other adult in the household often swear at you, insult you, put you down, or humiliate you? | 20.5 |
| Physical abuse | |  |
|  | Did a parent or other adult in the household ever hit you so hard that you had marks or were injured? | 14.5 |
|  | Did a parent or other adult in the household often push, grab, slap, or throw something at you? | 12.4 |
| Interpersonal violence | |  |
|  | Was your mother or stepmother sometimes kicked, bitten, hit with a fist, or hit with something hard? | 5.5 |
|  | Was your mother or stepmother often pushed, grabbed or slapped, or did she have something thrown at her? | 2.9 |
|  | Was your mother or stepmother ever repeatedly hit for at least a few minutes or threatened with a gun or knife? | 2.7 |
| Sexual abuse | |  |
|  | Did an adult or person at least 5 years older than you ever touch or fondle you or have you touch their body in a sexual way? | 1.5 |
|  | Did an adult or person at least 5 years older than you ever try to or have oral, anal, or vaginal sex with you? | 1.4 |
| **Deprivation** | |  |
| Emotional neglect | |  |
|  | Did you often feel that your family didn’t look out for each other, feel close to each other, or support each other? | 28.1 |
|  | Did you often feel that no one in your family loved you or thought you were important or special? | 13.3 |
|  | Did you often feel that you had no one to protect you? | 8.4 |
| Household dysfunction | |  |
|  | Were your parents ever separated or divorced? | 13.7 |
|  | Did you live with anyone who was a problem drinker or alcoholic or who used street drugs? | 9.8 |
|  | Was a household member depressed or mentally ill or did a household member attempt suicide? | 3.1 |
| Physical neglect | |  |
|  | Did you often feel that you didn’t have enough to eat? | 9.7 |
|  | Did a household member go to prison? | 7.6 |
|  | Did you often feel that your parents were too drunk or high to take care of you or take you to the doctor if you needed it? | 5.3 |
|  | Did you often feel that you had to wear dirty clothes? | 4.8 |

Appendix A

**Table A.1.** Questions included in the ACEs questionnaire and their prevalence in CLHNS. Questions were categorized into seven distinct categories from which cumulative ACEs was calculated, and then categories were binned into two dimensions of adversity to calculate threat and deprivation scores.

|  |  | **Threat ACEs** | | |  | **Deprivation ACEs** | | |
| --- | --- | --- | --- | --- | --- | --- | --- | --- |
| **Predictor** |  | **IRR**^†^ | **95% CI**^‡^ | **p-value** |  | **IRR**^†^ | **95% CI**^‡^ | **p-value** |
| Household assets |  | **1.09** | **[1.00, 1.19]** | **0.048** |  | 1.07 | [0.98, 1.17] | 0.119 |
| Maternal education |  | 1.08 | [0.99, 1.18] | 0.091 |  | 0.97 | [0.88, 1.06] | 0.461 |
| Male |  | **0.73** | **[0.59, 0.91]** | **0.005** |  | 0.97 | [0.78, 1.20] | 0.767 |
| ***ZI formula*** |  | **Coef.** | **95% CI**^‡^ | **p-value** |  | **Coef.** | **95% CI**^‡^ | **p-value** |
| Male |  | 1.44 | [0.86, 2.42] | 0.164 |  | **2.41** | **[1.42, 4.11]** | **0.001** |

**Table A.2.** Model output for generalized linear models (Poisson) predicting threat and deprivation scores from baseline SES variables and participant sex (assigned at birth). Participant sex was used as a predictor of zero-inflation (ZI). ^†^IRR = incidence rate ratio. ^‡^CI = confidence interval.

|  |  | **Model 1** | | |  | **Model 2** | | |
| --- | --- | --- | --- | --- | --- | --- | --- | --- |
| **Outcome** | **Predictor** | **Coef.** | **95% CI**^†^ | **p-value** |  | **Coef.** | **95% CI**^†^ | **p-value** |
| **Menarche** | Cumulative ACEs | 1.02 | [0.95, 1.09] | 0.591 |  |  |  |  |
|  | Threat score |  |  |  |  | 1.05 | [0.96, 1.16] | 0.296 |
|  | Deprivation score |  |  |  |  | 0.98 | [0.89, 1.09] | 0.758 |
|  | Household assets | 1.03 | [0.94, 1.12] | 0.562 |  | 1.03 | [0.93, 1.12] | 0.594 |
|  | Maternal edu | **1.21** | **[1.10, 1.32]** | **<0.001** |  | **1.20** | **[1.10, 1.32]** | **<0.001** |
|  | Maternal age at menarche | **0.85** | **[0.78, 0.93]** | **<0.001** |  | **0.85** | **[0.78, 0.92]** | **<0.001** |
| **First conception** | Cumulative ACEs | 1.07 | [1.00, 1.15] | 0.062 |  |  |  |  |
|  | Threat score |  |  |  |  | 1.04 | [0.94, 1.15] | 0.407 |
|  | Deprivation score |  |  |  |  | **1.11** | **[1.00, 1.23]** | **0.043** |
|  | Household assets | 0.94 | [0.85, 1.04] | 0.217 |  | 0.94 | [0.84, 1.04] | 0.204 |
|  | Maternal edu | **0.87** | **[0.79, 0.96]** | **0.004** |  | **0.86** | **[0.78, 0.95]** | **0.004** |
|  | Maternal age at menarche | 0.95 | [0.87, 1.03] | 0.227 |  | 0.95 | [0.86, 1.03] | 0.216 |
| **Gravidity** | Cumulative ACEs | **1.07** | **[1.01, 1.12]** | **0.012** |  |  |  |  |
|  | Threat score |  |  |  |  | 1.04 | [0.98, 1.10] | 0.233 |
|  | Deprivation score |  |  |  |  | 1.06 | [1.00, 1.12] | 0.051 |
|  | Household assets | 0.99 | [0.94, 1.05] | 0.706 |  | 0.99 | [0.93, 1.04] | 0.609 |
|  | Maternal edu | **0.89** | **[0.84, 0.94]** | **<0.001** |  | **0.89** | **[0.84, 0.94]** | **<0.001** |
|  | Maternal age at menarche | 1.01 | [0.95, 1.06] | 0.840 |  | 1.01 | [0.96, 1.06] | 0.782 |
|  | Age | 0.98 | [0.93, 1.03] | 0.428 |  | 0.98 | [0.93, 1.03] | 0.430 |

**Table A.3.**  Results from the adjusted models predicting biodemographic life history traits in females. Discrete hazard models (Gompertz) were run for menarche and first conception in females, and the coefficients reflect the hazard ratio (HR). Generalized linear models (zero-inflated Poisson) were run for gravidity and the coefficient reflects the incidence rate ratio. ^†^CI = confidence interval.

|  |  | **Model 1** | | |  | **Model 2** | | |
| --- | --- | --- | --- | --- | --- | --- | --- | --- |
| **Outcome** | **Predictor** | **Coef.** | **95% CI** | **p-value** |  | **Coef.** | **95% CI** | **p-value** |
| **Early puberty** | Cumulative ACEs | 1.15 | [0.98, 1.34] | 0.087 |  |  | | |
|  | Threat score |  |  |  |  | 1.11 | [0.94, 1.31] | 0.233 |
|  | Deprivation score |  |  |  |  | 1.03 | [0.86, 1.21] | 0.772 |
|  | Household assets | 1.16 | [0.97, 1.39] | 0.102 |  | 1.16 | [0.97, 1.39] | 0.102 |
|  | Maternal edu | **1.36** | **[1.14, 1.63]** | **<0.001** |  | **1.36** | **[1.14, 1.63]** | **<0.001** |
|  | Maternal age at menarche | 0.88 | [0.75, 1.03] | 0.111 |  | 0.88 | [0.75, 1.03] | 0.112 |
|  | Age | **1.29** | **[1.11, 1.52]** | **0.001** |  | **1.29** | **[1.10, 1.52]** | **0.002** |

**Table A.4.** Results from the adjusted models predicting advanced pubertal development in males. Generalized linear models were run with a binomial distribution and the coefficient reflects the odds ratio. CI = confidence interval.

|  |  | **Model 1** | | |  | **Model 2** | | |
| --- | --- | --- | --- | --- | --- | --- | --- | --- |
| **Outcome** | **Predictor** | **HR**^†^ | **95% CI**^‡^ | **p-value** |  | **HR**^†^ | **95% CI**^‡^ | **p-value** |
| **First smoke** | Cumulative ACEs | **1.16** | **[1.04, 1.30]** | **0.007** |  |  |  |  |
|  | Threat score |  |  |  |  | **1.20** | **[1.04, 1.39]** | **0.012** |
|  | Deprivation score |  |  |  |  | 1.09 | [0.93, 1.26] | 0.277 |
|  | Household assets | 1.05 | [0.92, 1.20] | 0.475 |  | 1.03 | [0.90, 1.18] | 0.644 |
|  | Maternal edu | 1.13 | [0.98, 1.30] | 0.086 |  | 1.12 | [0.98, 1.29] | 0.102 |
|  | Maternal age at menarche | 1.01 | [0.88, 1.15] | 0.908 |  | 1.01 | [0.88, 1.15] | 0.929 |
| **First drink** | Cumulative ACEs | 1.04 | [0.96, 1.11] | 0.331 |  |  |  |  |
|  | Threat score |  |  |  |  | 1.08 | [0.98, 1.19] | 0.109 |
|  | Deprivation score |  |  |  |  | 0.96 | [0.87, 1.06] | 0.419 |
|  | Household assets | 1.01 | [0.92, 1.11] | 0.800 |  | 1.01 | [0.92, 1.11] | 0.804 |
|  | Maternal edu | **1.12** | **[1.02, 1.22]** | **0.018** |  | **1.11** | **[1.01, 1.22]** | **0.023** |
|  | Maternal age at menarche | 1.04 | [0.96, 1.13] | 0.335 |  | 1.04 | [0.96, 1.13] | 0.367 |
| **First relationship** | Cumulative ACEs | 1.07 | [0.99, 1.14] | 0.073 |  |  |  |  |
|  | Threat score |  |  |  |  | 1.07 | [0.98, 1.17] | 0.138 |
|  | Deprivation score |  |  |  |  | 1.05 | [0.95, 1.15] | 0.327 |
|  | Household assets | 1.04 | [0.94, 1.14] | 0.463 |  | 1.03 | [0.94, 1.13] | 0.534 |
|  | Maternal edu | **0.88** | **[0.80, 0.96]** | **0.006** |  | **0.88** | **[0.80, 0.96]** | **0.006** |
|  | Maternal age at menarche | 0.95 | [0.88, 1.03] | 0.220 |  | 0.95 | [0.88, 1.03] | 0.230 |
| **Sexual debut** | Cumulative ACEs | **1.12** | **[1.04, 1.20]** | **0.001** |  |  |  |  |
|  | Threat score |  |  |  |  | 1.07 | [0.98, 1.18] | 0.141 |
|  | Deprivation score |  |  |  |  | **1.13** | **[1.03, 1.25]** | **0.012** |
|  | Household assets | 0.95 | [0.87, 1.05] | 0.333 |  | 0.95 | [0.86, 1.04] | 0.294 |
|  | Maternal edu | **0.90** | **[0.82, 0.98]** | **0.022** |  | **0.90** | **[0.82, 0.98]** | **0.018** |
|  | Maternal age at menarche | 0.97 | [0.90, 1.06] | 0.534 |  | 0.97 | [0.89, 1.06] | 0.499 |

**Table A.5.**  Results from the adjusted models predicting the onset of behavioral life history-related traits in females. Discrete hazard models (Gompertz) were run for each outcome of interest. ^†^HR = hazard ratio. ^‡^CI = confidence interval.

|  |  | **Model 1** | | |  | **Model 2** | | |
| --- | --- | --- | --- | --- | --- | --- | --- | --- |
| **Outcome** | **Predictor** | **HR**^†^ | **95% CI**^‡^ | **p-value** |  | **HR**^†^ | **95% CI**^‡^ | **p-value** |
| **First smoke** | Cumulative ACEs | 1.06 | [0.98, 1.14] | 0.130 |  |  |  |  |
|  | Threat score |  |  |  |  | 1.05 | [0.94, 1.17] | 0.353 |
|  | Deprivation score |  |  |  |  | 1.04 | [0.94, 1.15] | 0.419 |
|  | Household assets | 1.03 | [0.94, 1.13] | 0.506 |  | 1.03 | [0.94, 1.13] | 0.504 |
|  | Maternal edu | **0.88** | **[0.80, 0.97]** | **0.007** |  | **0.88** | **[0.80, 0.97]** | **0.007** |
|  | Maternal age at menarche | 1.06 | [0.98, 1.15] | 0.153 |  | 1.01 | [0.88, 1.15] | 0.929 |
| **First drink** | Cumulative ACEs | **1.10** | **[1.03, 1.18]** | **0.006** |  |  |  |  |
|  | Threat score |  |  |  |  | 1.01 | [0.91, 1.12] | 0.835 |
|  | Deprivation score |  |  |  |  | **1.12** | **[1.01, 1.23]** | **0.031** |
|  | Household assets | 1.04 | [0.95, 1.13] | 0.403 |  | 1.04 | [0.95, 1.13] | 0.377 |
|  | Maternal edu | 0.97 | [0.89, 1.05] | 0.437 |  | 0.97 | [0.89, 1.06] | 0.516 |
|  | Maternal age at menarche | 1.01 | [0.94, 1.09] | 0.739 |  | 1.01 | [0.94, 1.09] | 0.722 |
| **First relationship** | Cumulative ACEs | 1.02 | [0.95, 1.10] | 0.516 |  |  |  |  |
|  | Threat score |  |  |  |  | 0.97 | [0.87, 1.08] | 0.613 |
|  | Deprivation score |  |  |  |  | 1.08 | [0.98, 1.19] | 0.108 |
|  | Household assets | 1.04 | [0.95, 1.13] | 0.444 |  | 1.03 | [0.94, 1.13] | 0.468 |
|  | Maternal edu | 1.03 | [0.94, 1.12] | 0.527 |  | 1.03 | [0.95, 1.13] | 0.468 |
|  | Maternal age at menarche | 0.95 | [0.88, 1.02] | 0.145 |  | 0.95 | [0.88, 1.02] | 0.143 |
| **Sexual debut** | Cumulative ACEs | 1.01 | [0.94, 1.09] | 0.747 |  |  |  |  |
|  | Threat score |  |  |  |  | 0.94 | [0.84, 1.04] | 0.206 |
|  | Deprivation score |  |  |  |  | **1.12** | **[1.01, 1.23]** | **0.027** |
|  | Household assets | 0.98 | [0.89, 1.07] | 0.627 |  | 0.97 | [0.89, 1.06] | 0.560 |
|  | Maternal edu | **1.10** | **[1.01, 1.20]** | **0.034** |  | **1.11** | **[1.01, 1.20]** | **0.023** |
|  | Maternal age at menarche | 1.06 | [0.98, 1.14] | 0.135 |  | 1.06 | [0.98, 1.14] | 0.124 |

**Table A.6.**  Results from the adjusted models predicting the onset of behavioral life history-related traits in males. Discrete hazard models (Gompertz) were run for each outcome of interest. ^†^HR = hazard ratio. ^‡^CI = confidence interval.


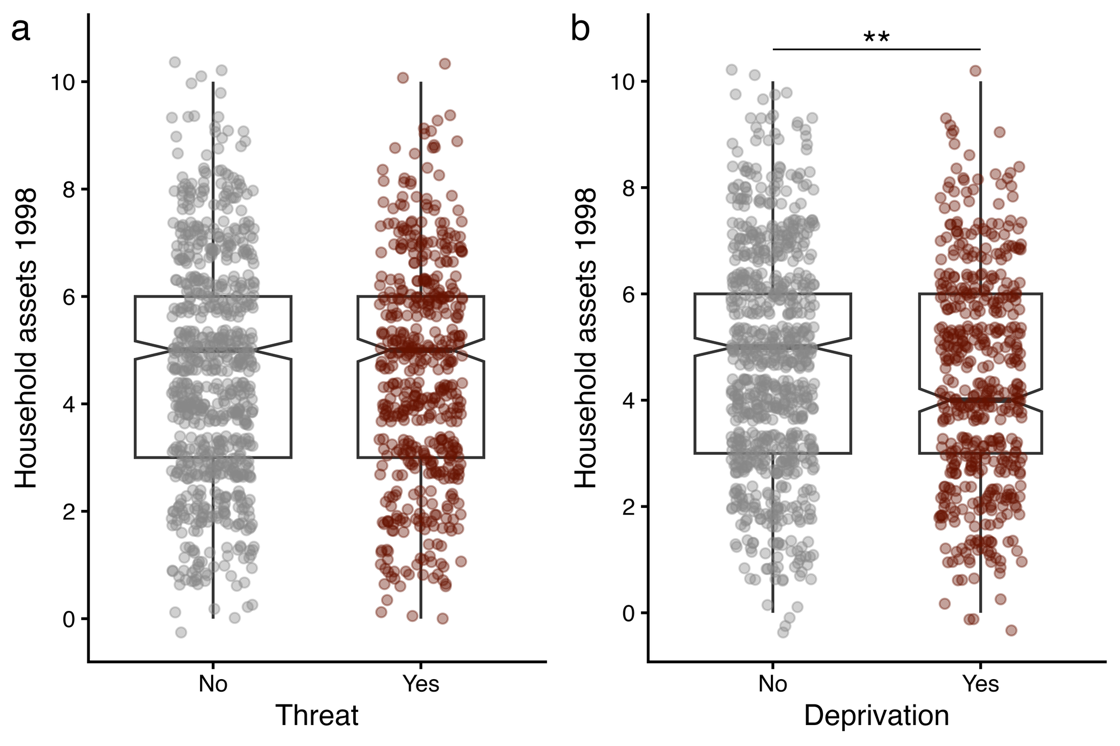


**Figure A.1. Socioeconomic status in adolescence and whether an individual reported ACEs related to (a) threat or (b) deprivation.** p-values are from a one-sided Wilcoxon signed rank test based on the prediction that individuals exposed to ACEs would be from households of lower SES.


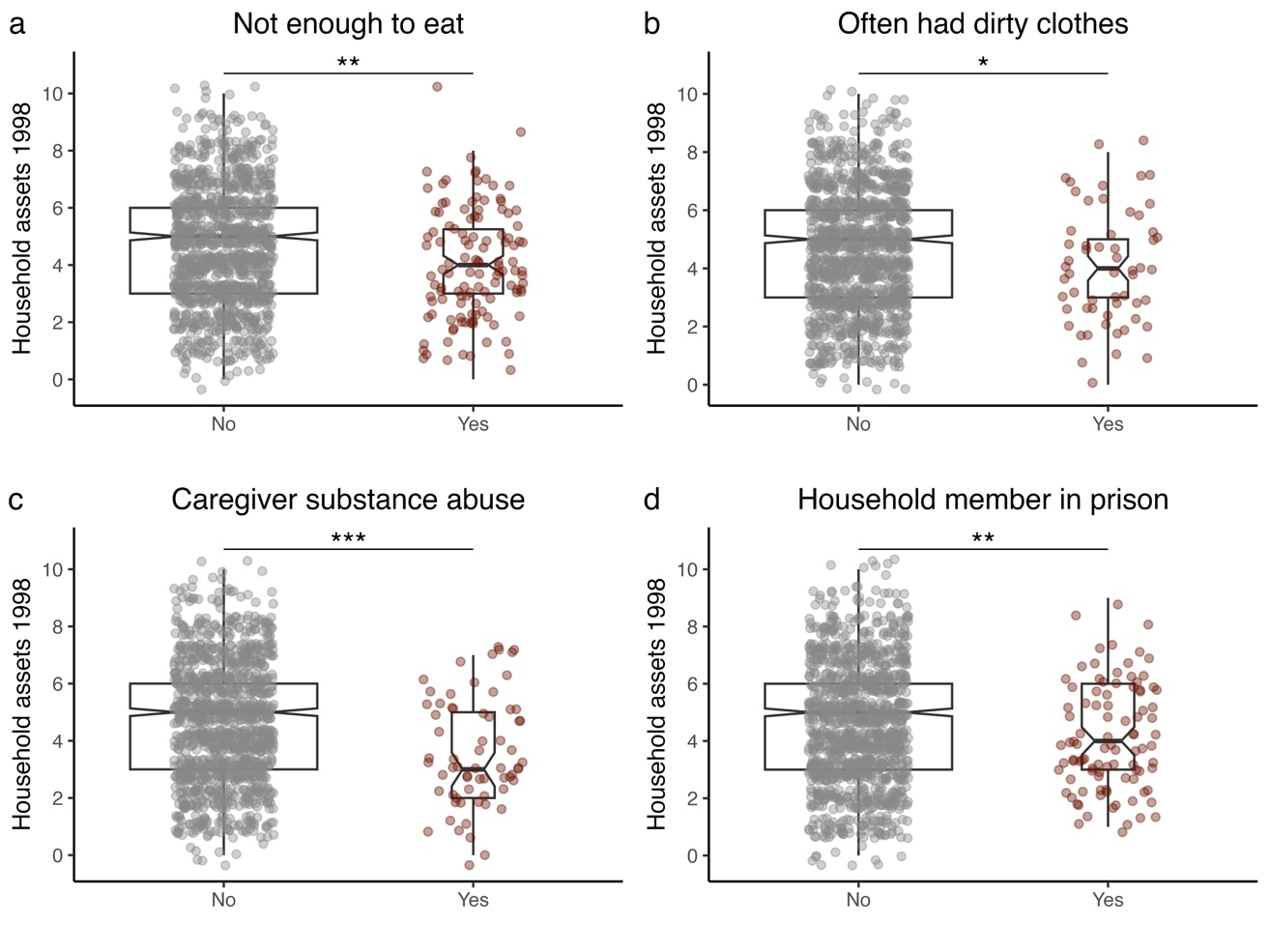


**Figure A.2. Socioeconomic status in adolescence and whether an individual reported experiencing physical neglect.** (a) Often felt that they did not have enough to eat, (b) often felt that they had to wear dirty clothes, (c) often felt that their parents were too drunk or high to care for them, (d) had a household member go to prison. p-values are from one-sided Wilcoxon signed rank tests based on the prediction that individuals exposed to physical neglect would be from households of lower SES.


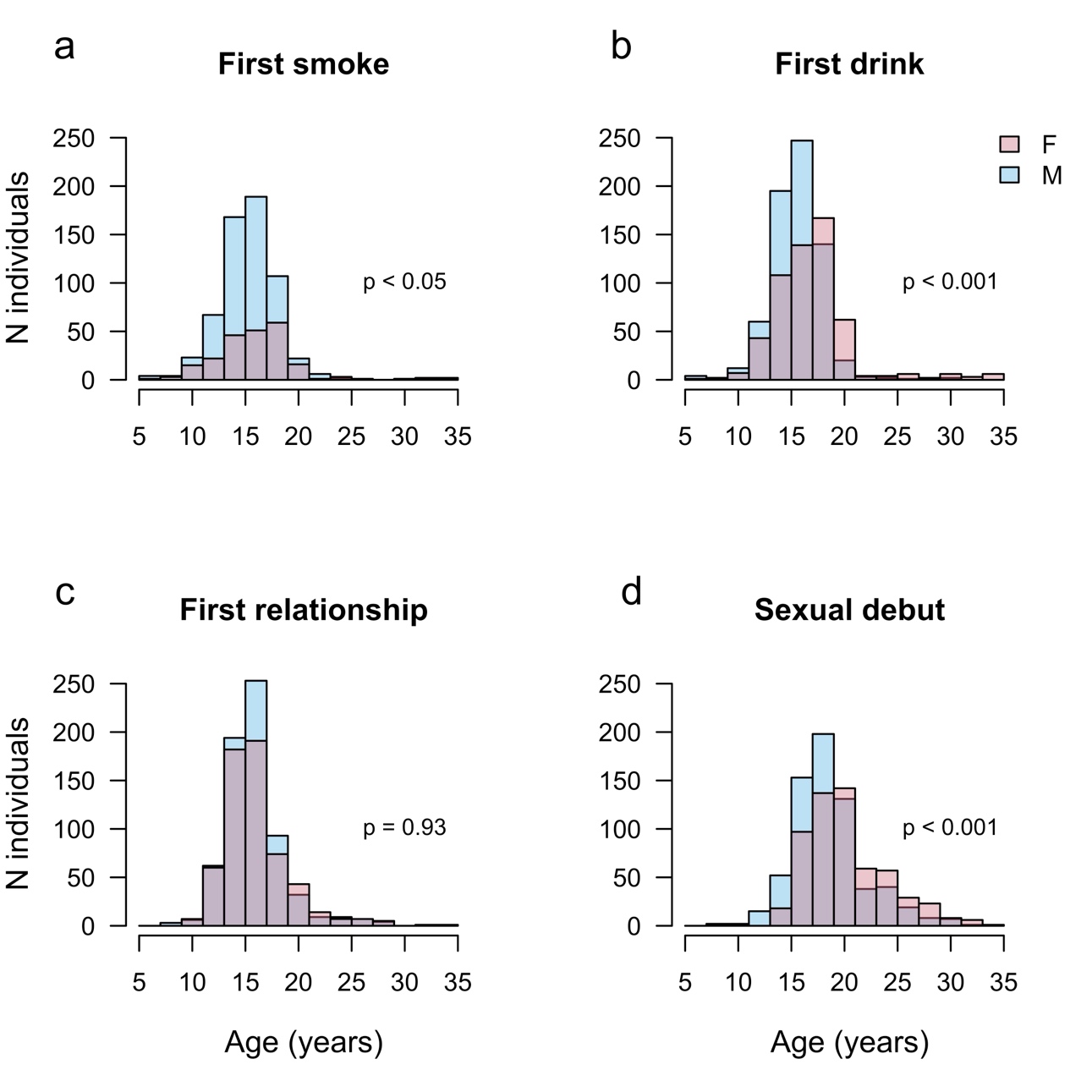


**Figure A.3. Distributions of the age at onset for risk-taking behaviors females (pink) and males (blue).** (a) first smoke, (b) first drink, (c) first romantic relationship, and (d) sexual debut. p-values are from two-sided Wilcoxon signed rank tests.
